# Case Report: Nutrition profile, body mass, sleep quality, and acute mountain sickness symptoms during altitude training in a Paralympic record-breaking athlete and his two guides

**DOI:** 10.3389/fnut.2025.1706179

**Published:** 2025-12-04

**Authors:** Kimberly Belluco, Fabio Leandro Breda, Thiago Fernando Lourenço, Carolina Cirino, Marcelo Papoti, Claudio Alexandre Gobatto, Fúlvia Barros Manchado-Gobatto

**Affiliations:** 1 School of Applied Sciences, University of Campinas (UNICAMP), Limeira, São Paulo, Brazil; 2 Hypoxia, Sport, and Health Team, University of Campinas (UNICAMP), Limeira, São Paulo, Brazil; 3 Brazilian Paralympic Committee (CPB/SP), São Paulo, Brazil; 4 School of Physical Education and Sport (USP), Ribeirão Preto, Brazil

**Keywords:** nutrition, food consumption, altitude, sports performance, mountain sickness, sleep

## Abstract

Altitude training under the “live high-train high” regime is commonly used by athletes to enhance sea-level performance. However, careful monitoring is required, since nutritional aspects, sleep quality, and acclimatization symptoms may influence results and pose health risks. This is especially relevant in elite athletes aiming for peak performance. The aim of this case study was to assess the nutritional profile, body mass, sleep quality, and mountain sickness symptoms of a Paralympic athletics world record holder in the T11 class (1A), three-time world record holder, and his two guides (2G and 3G), with weekly measurements during the altitude training period. The athletes underwent 30 days of training in Colombia, in a city located approximately 2,150 meters above sea level. At baseline and during each training week, body weight, a food recall, and symptoms of Acute Mountain Sickness (AMS) were collected using the Lake Louise Symptom Score. Sleep quality was analyzed twice, before and after training, using the Epworth scale (ESS) and the Pittsburgh questionnaire (PSQI). No major changes were observed in body mass (1A: 1.52%; 2G: −2.67%; 3G: 0.16%) or sleep quality, which remained stable across both assessments. On the ESS, 2G showed scores indicative of excessive daytime sleepiness, while on the PSQI, 3G demonstrated suboptimal sleep quality; values did not change pre- to post-altitude. Mild AMS was reported by 1A and 2G on day 1 (scores 3–5), whereas 3G showed no symptoms. Appetite remained stable, but carbohydrate intake (1A: 4.8–5.7 g·kg^−1^·day^−1^; 2G: 3.8–5.9 g·kg^−1^·day^−1^; 3G: 4.4–5.4 g·kg^−1^·day^−1^) was below American College of Sports Medicine (ACSM) recommendations. These findings underscore the importance of systematically monitoring nutrition, body mass, sleep quality, and AMS symptoms during altitude training, providing practical guidance for coaches and supporting optimal preparation of Paralympic athletes.

## 1 Introduction

Altitude training has been recognized as an effective strategy for improving athletic performance. Exposure to hypoxic environmental conditions can induce morphological and physiological adaptations that potentially influence performance at sea level (1–4). This training can be conducted at real altitudes in locations with moderate or high elevations or simulated conditions using hypoxic tents or auto-hypoxicators. There are several protocols for conducting altitude training, including: Live High-Train High (LHTH), Live Low-Train High (LLTH), Live High-Train Low (LHTL), and Intermittent Hypoxia (IHT) (5). The most common model used by athletes is the LHTH model (4), which consists of living and training at altitude for two to four weeks to obtain acclimatization-related adaptations, such as improved aerobic capacity resulting from increased maximum oxygen volume (VO₂ max), hemoglobin content, and erythropoietin concentration (4, 6, 7).

A recent study conducted by our group with high-performance long-distance and middle-distance athletes revealed a decline in performance related to aerobic capacity during the first six days after returning from altitude training on the LHTH regimen. However, fifteen days after returning to low altitude (approximately 760 m at sea level), the athletes achieving positive performance. These results suggest that physiological adaptations to altitude require a brief recovery period at sea level to manifest fully (8).

Exposure to environmental conditions at moderate and high altitudes can lead to changes in body composition, energy expenditure, eating habits (9–11), the appearance of symptoms of Acute Mountain Sickness (AMS) in non-acclimatized individuals (12) and sleep quality (13, 14). These changes can compromise the sports performance when not effectively controlled. It has been observed that altitude training may lead to appetite loss, difficulty in consuming sufficient amounts of food, and gastrointestinal disturbances, which can impair nutrient absorption and hinder the attainment of total energy expenditure, resulting in weight loss (15). In this context, ensuring adequate caloric intake is a priority to prevent low energy availability, which may result in performance decline, increased injury risk, and greater iron requirements that may result in micronutrient deficiencies (9). Therefore, a personalized nutritional plan that meets the specific needs of athletes exposed to altitude training, particularly Paralympic athletes, is essential (11).

In addition to nutritional factors, recovery-related aspects such as sleep also play a critical role in adaptation to hypoxic environments. Sleep quality may be compromised due to reduced oxygen saturation and disruptions in circadian regulation, potentially impairing recovery, and adaptation to training (16). Indeed, one study reported a ~ 3% decline in sleep quality under hypoxic conditions (14). Conversely, Olympic-level open-water swimmers exposed to 14 days of altitude training did not exhibit significant changes in all objective or subjective sleep quality parameters, suggesting that monitoring sleep during hypoxic exposure is valuable to ensure that sleep disturbances do not undermine the benefits of altitude training (13).

AMS, also known as altitude sickness, is considered a subjective syndrome because it presents with nonspecific symptoms such as nausea, headache, gastrointestinal symptoms, dizziness, tiredness, and fatigue. These symptoms are usually triggered six to ten hours after ascending to high altitudes (17). It is typically triggered in individuals who are not acclimatized and have been exposed to an altitude above 2,500 meters (m), and it is rarely triggered at altitudes below this (17, 18). Therefore, if an athlete experiences these symptoms, they should seek medical treatment and stop climbing, if possible. If symptoms do not subside, returning to a lower altitude may be necessary (3). Therefore, it is extremely important to evaluate and control these parameters in athletes who are exposed to environmental hypoxia conditions so that they can maximize the benefits of this training while minimizing the risks or deleterious effects. However, there are gaps in the literature on this topic, particularly regarding high-performance athletes (19).

Researching the LHTH model is no simple task, especially in the context of Olympic sport. Daily monitoring of athletes by multidisciplinary teams at altitude is necessary, as is respect for the scientific method, combined with a greater concern for maximizing the performance of these elite athletes (20). This is even more challenging in the context of Paralympic sport, as was the case study which investigated a visually impaired Paralympic athlete who had set world records on several occasions in middle-distance events, including being a three-time world record holder, twice in the 1,500 meters (m) and once in the 5,000 m. In this regard, it is important to recognize that visually impaired athletes may experience compromised sleep quality and impaired recovery, as diminished light perception can disrupt circadian rhythms and melatonin secretion (21). Moreover, these athletes may exhibit a slightly lower resting metabolic rate, subtle deficits in motor development and rhythm that can hinder the acquisition of sport-specific techniques, and challenges in effectively monitoring hydration and energy intake (22).

Thus, the purpose of this case study was to assess the nutritional profile, body mass, sleep quality and symptoms of AMS of a Paralympic record holder athlete and his two guides during a four-week moderate altitude training period. Specifically, it aimed to monitor and investigate the effect of hypoxia associated to physical training (LHTH) at 2,150 m on food consumption, body mass and symptoms of AMS, with weekly measurements during the altitude training period and the quality of sleep of athletes subjected to 30 days of exposure to moderate altitude training.

## 2 Case description and assessment methods

### 2.1 Case presentation

A male Paralympic long-distance runner (1A = 30 y, 1.79 m, 64 kg) in the T11 class (visual acuity of less than LogMAR 2.60) and his two male guide runners (2G = 30 y, 1.74 m, 64 kg; 3G = 33 y, 1.84 m, 81 kg), who do not have any disabilities, underwent 30 days of altitude training in preparation for future competitions. The T11 class is designated for athletes who are totally blind or have a severe visual impairment. They must compete with a guide using a tether and are required to wear a blindfold or opaque glasses (23). The case athlete’s visual impairment is associated with a congenital retinal condition that was diagnosed in childhood and has progressively worsened over time, known as a congenital retinal disorder.

The chose for this case study based on the Paralympic athlete outstanding achievements in World championships and the Paralympic Games, as well as his thoroughly monitored training at real altitude. For example, this athlete is a Paralympic champion and World record holder in both the 1,500 m and 5,000 m events, having claimed the titles at the Tokyo 2020 + 1 Paralympic Games and the 2024 Kobe World Athletics Championships. He also won the world title in the 1,500 m in Paris 2023 and set the world record for the event at the 2024 Kobe World Championships. Recently, this athlete won a gold medal at the New Delhi 2025 World Para athletes Championship. Regarding his guides, although they are not officially recognized as record holders due to competitions regulations, their contribution is essential to the visually impaired athlete’s performance. To effectively accompany and support him during training and competition, the guides must possess exceptional physical, physiological, and running profile.

According to the anamnesis, the athlete and his two guides were professional athletes with completed secondary education. At the time of data collection, the athlete (1A) and one guide (3G) were enrolled in undergraduate programs. They had no food allergies or intolerances, did not use continuous medication, had no pathologies. They also did not present dental issues or problems interfering with food consumption, reported between 7 and 8 hours of sleep per day, did not have a smoking habit, and exhibited regular bowel habits. Nutritional monitoring was reported by only 1A and 2G, with 2G also reporting alcohol consumption twice a week, an aversion to vegetables, and a weight loss of 2 kilograms over the previous 2 months, corresponding to a 3.03% reduction in total body mass. 1A and 3G were married and lived with their spouses. They ate meals prepared by their wives at home. In contrast, 2G lived with another athlete and ate outside the home. Additionally, only 1A reported a family history of diseases such as hypertension, mentioning his mother.

Three athletes provided written consent, and the study was approved by the Research Ethics Committee of the Faculty of Medical Sciences, UNICAMP, Protocol No. CAAE 52313721.0.0000.5404.

### 2.2 Altitude training

The altitude training protocol was executed in Guarne city, Colombia, at approximately 2,150 m above sea level, with duration of 30 days. During this period, the athletes and technical team lived in the same residence, which facilitated control over nutrition, hydration, and recovery after training sessions. The program comprised weekly seven-day microcycles, encompassing a total of 11 training sessions. These sessions included two high-intensity interval training sessions, two strength training sessions in the gym, one plyometric session, one high-volume session, one flexibility session, and four regenerative sessions. The total weekly volume exhibited a range from 110 to 140 km. The athletes’ physiological acclimatization period and load progression were respected with adjustments in volume and intensity. The intensity of the training was individualized based on pre-altitude aerobic and anaerobic assessments, which allowed classification into three zones: zone 1 (Z1), considered low intensity, defined as a running pace below or at ventilatory threshold (vVT); zone 2 (Z2), defined as a pace between the vVT and respiratory compensation point velocity (vRCP) 2; and zone 3 (Z3), at or above the vRCP. These zones were used to guide the training periodization (8).

In the first week, the total training volume was 110 km, with 80 km performed at an intensity close to Z1, corresponding to the first ventilatory threshold (VT1), and 30 km in Z2, between VT1 and the respiratory compensation point (VT2). As part of the adaptation process to the effects of hypoxic exposure, both the training volume and intensity progressively increased over the following weeks. By the fourth week, the athletes completed 140 km of training, consisting of 90 km near Z1, 30 km within Z2, and 20 km in Z3. The athletes exhibited very similar physical conditioning, so the training sessions did not differ among them. The duration of the sessions, however, varied according to the training objective. Regenerative sessions at an easy pace lasted approximately 30 to 45 min, continuous endurance sessions ranged from 60 to 80 min, and interval sessions could last up to 120 min, including warm-up, main set, and cool-down. Strength training sessions lasted about 100 min, including warm-up. Strength training sessions were performed using free weights, with athletes completing three sets of 10 to 16 repetitions per exercise. The load was determined according to perceived exertion, aiming to reach near muscle failure in the final repetition of each set. Throughout the entire training period, all sessions were supervised by a coach specialized in Paralympic athletics.

### 2.3 Dietary intake

Prior to departure for Colombia, the athlete and his guides underwent a comprehensive nutritional assessment and completed a food frequency questionnaire. The goal was to better characterize the sample, understand their socioeconomic profile, food preferences, and dietary habits. A 24-h Food Recall (24) was used to assess intake before and during the four-week training period, with data collected at five time points: one week before the program and weekly during training program. The recalls were conducted through direct interviews by the athletes’ personal coach, who had been previously trained by a sports nutritionist for this procedure and accompanied them throughout the entire training period. Given evidence that elite endurance athletes self-report consistent dietary practices across training cycles (25), weekly 24-h recalls were considered sufficient to capture representative dietary intake.

Basal metabolic rate (BMR) was estimated for each training participant using the Harris-Benedict equation (26). These values are presented as approximate estimates of individual energy expenditure. Total energy expenditure (TEE) was then calculated by applying a physical activity factor of 1.725 to the estimated BMR. Energy intake was assessed using 24-h dietary recalls and compared to the estimated TEE to determine whether training participants met the recommended requirements for high-performance athletes during the altitude training period. The data were analyzed by the sports nutritionist using Diet Box software version 8.1.6 to estimate energy expenditure and nutrient intake, and subsequently compared with recommendations for high-performance athletes.

### 2.4 Body mass assessment

Body mass was assessed at baseline using a Sanitas SBF 14 Max portable scale (100 g sensitivity), with measurements recorded three times per week. This parameter was collected in a standardized manner, with consistent timing and location (in the morning, with a maximum variation of 1 hour), and in a fasting state with minimal clothing.

### 2.5 Acute mountain sickness assessment

The Lake Louise Symptom Score (LLSS) (12) was utilized to evaluate the presence of acute AMS, which is typified by the manifestation of headache, gastrointestinal discomfort (nausea), fatigue, and dizziness. Each symptom was evaluated on a scale ranging from 0 (absent) to 3 (severe/incapacitating). The total score was determined by summing the ratings for all four symptoms. The total score ranged from 3 to 5, indicating mild AMS; 6 to 9, moderate AMS; and 10 to 12, severe AMS. The presence of headache (a minimum of 1 point) was a mandatory criterion for AMS diagnosis. In order to circumvent the potential for confounding of travel-related symptoms, the LLSS was administered a minimum of 6 h after altitude exposure (12, 17). The LLSS was administered a total of eight times: six times at rest (on days 1, 2, and 3; and on the first day of weeks 2, 3, and 4), and twice post-exercise (4 h after training sessions at Zone 1 [low intensity, TZ1] and Zone 3 [severe intensity, TZ3]). These administrations were in accordance with the coach’s training protocol.

### 2.6 Sleep quality assessment

**Figure 1:**
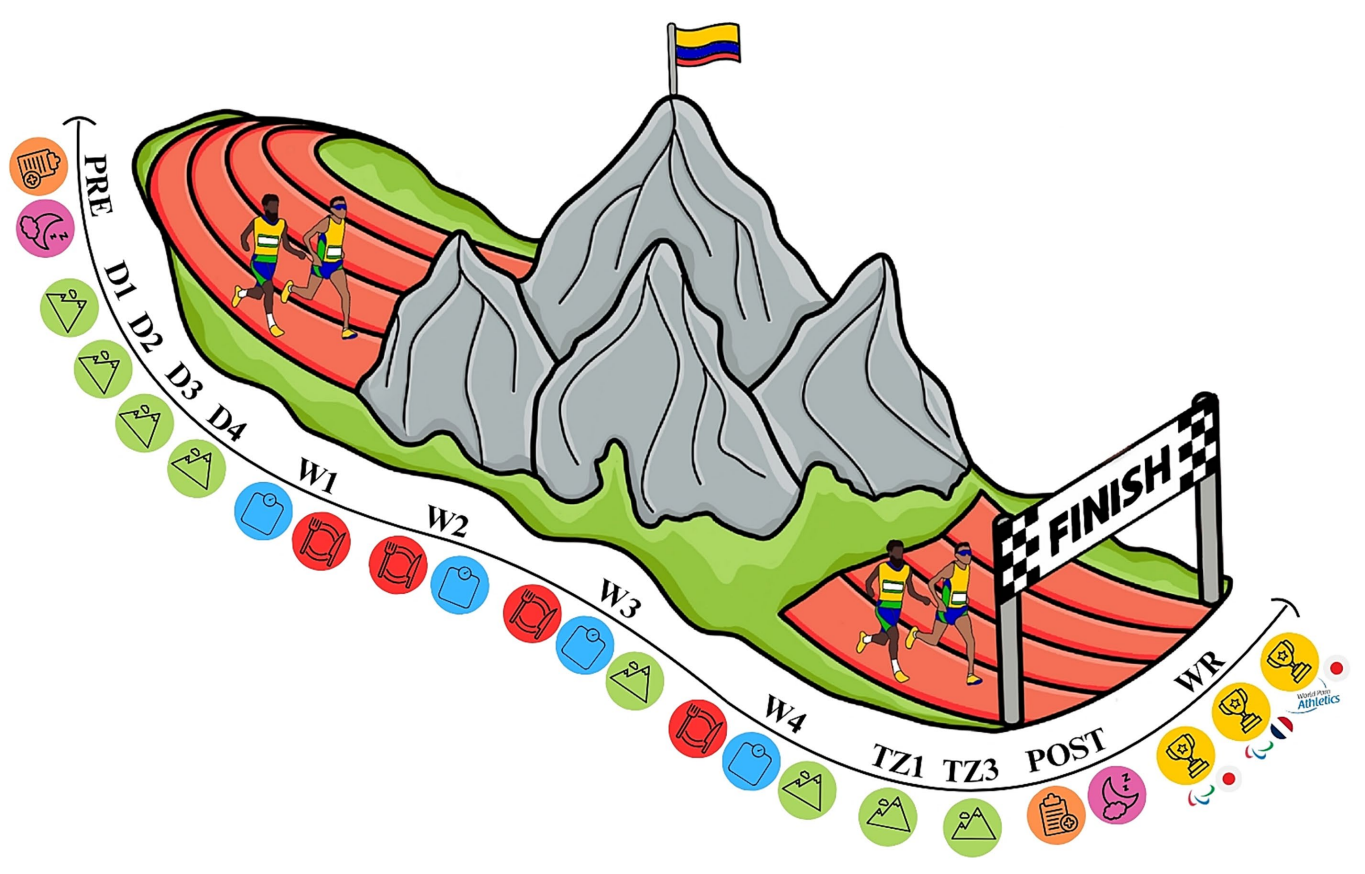
Timeline of observations. The timeline illustrates the key study phases: pre-altitude (PRE), day 1 (D1), day 2 (D2), day 3 (D3), day 4 (D4), week 1 (W1), week 2 (W2), week 3 (W3), week 4 (W4), training sessions in zone 1 (TZ1) and zone 3 (TZ3), post-altitude (POST), and world record (WR) performances. Training included endurance, high-intensity interval, strength, plyometric, flexibility, and regenerative sessions. Assessments of body mass, dietary intake, sleep quality, and acute mountain sickness symptoms were conducted before, during, and after the four-week altitude training period.

The evaluation of sleep was assessed using two validated instruments: the Pittsburgh Sleep Quality Index (PSQI) (27) and the Epworth Sleepiness Scale (ESS) (28). The PSQI comprises 19 self-reported items grouped into seven components, an additional five items optionally answered by a roommate, assessing sleep quality and disturbances over the past month. These questions are grouped into seven components: subjective sleep quality, sleep latency, sleep duration, habitual sleep efficiency, sleep disturbances, use of sleeping medications, and daytime dysfunction, generating a total score ranging from 0 to 21 points. A score between 0 and 4 indicates good sleep quality, scores between 5 and 10 indicate poor sleep quality, and scores above 10 represent the cutoff for sleep disorders (27). The ESS is a tool designed to evaluate the likelihood of dozing off in eight common daily situations, with a score ranging from 0 (no chance) to 3 (high chance). The total score ranges from 0 to 24, with values greater than 10 indicating excessive daytime sleepiness and values greater than 16 indicating severe sleepiness (28). Both questionnaires were self-administered at two distinct time points baselines (prior to altitude training) and post-altitude training (after 4 weeks of altitude exposure). The findings were then compared with established reference values from the literature to provide a context for interpretation (Figure 1).

## 3 Observations and outcomes

**Figure 2:**
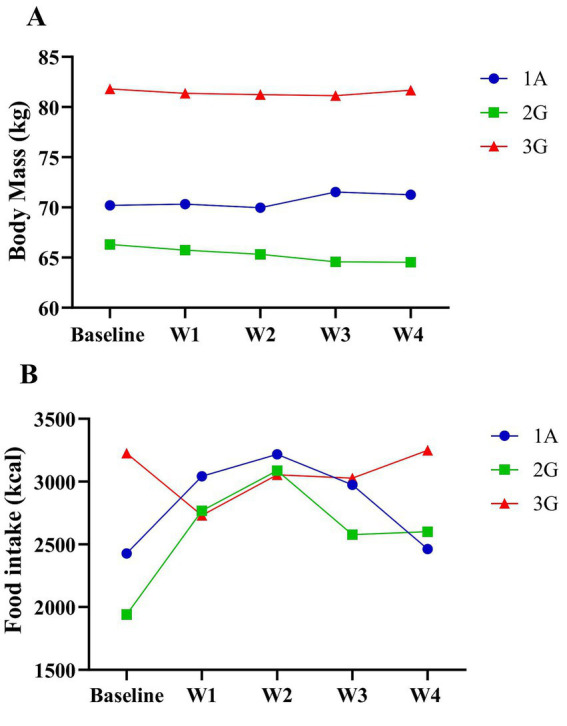
**(A)** Average weekly body mass of the athlete and his guides in kilograms (kg), based on weighing three times per week. **(B)** Total daily energy intake of the athlete and his guides, based on 24-h recall, expressed in kilocalories (kcal).

As illustrated in Figure 2A, the weekly average body mass of the athlete and his two guides is presented. The athlete (1A) showed a 1.52% increase in body mass, from 70.2 kg pre-altitude to 71.3 kg post-altitude. Guide 2 (2G) demonstrated a 2.67% reduction, decreasing from 66.3 kg to 64.5 kg, while Guide 3 (3G) exhibited a minimal change of 0.16%, from 81.8 kg to 81.7 kg. These intra-individual variations indicate that, despite minor fluctuations, body mass remained relatively stable throughout altitude exposure. Figure 2B illustrates the energy intake of the athlete and guides. During the first 2 weeks, 1A and 2G increased their energy intake from approximately 2,500 to 3,100 kcal and 2,000 to 2,800 kcal, respectively, while 3G initially decreased to about 2,400 kcal before gradually increasing, returning to around 3,000 kcal by week 4.

Basal metabolic rate (BMR) was estimated using the Harris-Benedict equation (26). The resulting values were 1714 kcal for 1A, 1872 kcal for 2G, and 1,589 kcal for 3G. Total energy expenditure was calculated by applying a physical activity factor of 1.725, resulting in 2957 kcal (1A), 3,229 kcal (2G), and 2,741 kcal (3G). Energy intake met the estimated requirements for 1A during weeks 1 to 3 and for 2G during weeks 1 and 2. For 3G, intake aligned with recommendations during the pre-altitude phase and week 4.

**Figure 3:**
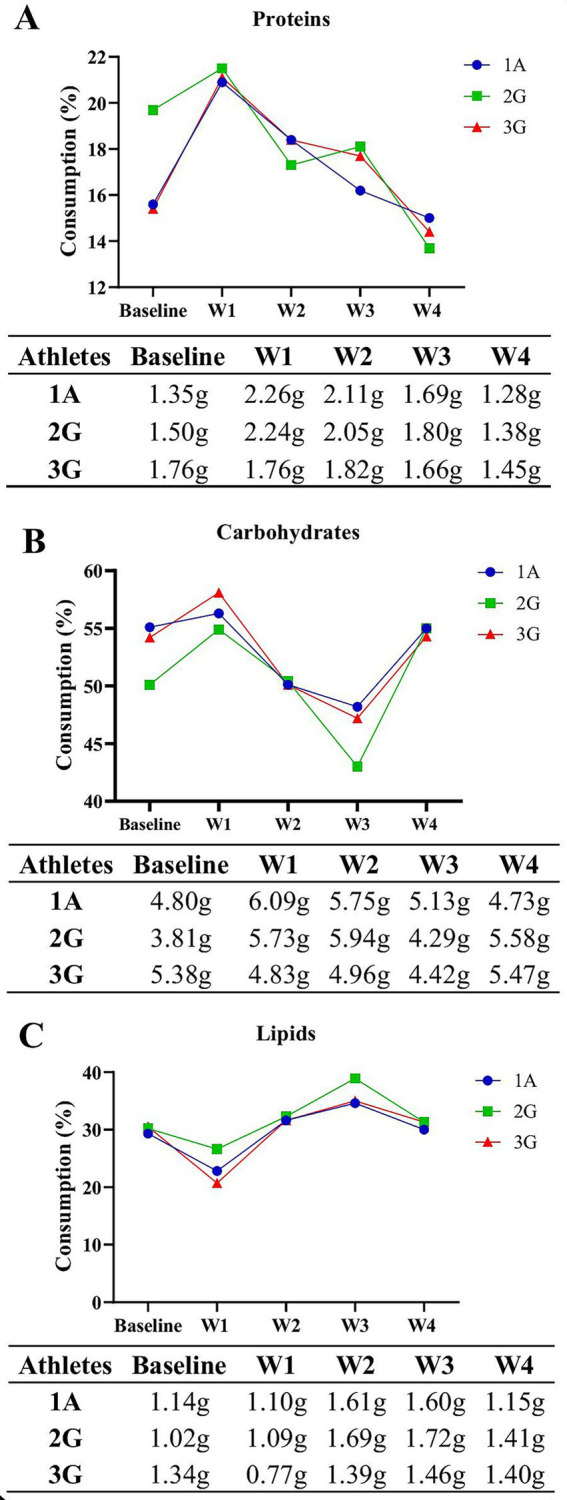
**(A)** Total carbohydrate intake (%, g·kg^−1^·day^−1^), obtained from the 24-h recall. **(B)** Total protein intake (%, g·kg^−1^·day^−1^), obtained from the 24-h recall. **(C)** Total lipid intake (%, g·kg^−1^·day^−1^), obtained from the 24-h recall.

According to the American College of Sports Medicine (ACSM) guidelines (6 g·kg^−1^·day^−1^), the recommended carbohydrate intake was 420 g for 1A, 384 g for 2G, and 486 g for 3G, consistent with moderate to lower-end high-intensity activity recommendations (29). As shown in Figure 3A, carbohydrate intake increased during the first week, declined in weeks 2 and 3, and rose again in week 4 relative to baseline. When adjusted for body weight, carbohydrate intake before altitude training (baseline) was 4.80, 3.81, and 5.38 g·kg^−1^·day^−1^ for athletes 1A, 2G, and 3G, respectively, remaining below the recommended levels throughout the training period.

Figure 3B shows that all athletes and guides met or exceeded the ACSM’s recommended daily protein intake for athletes (1.2–2.0 g·kg^−1^·day^−1^) (29). Regarding lipid intake, the recommendations established by the International Society of Sports Nutrition (ISSN) and the American Dietetic Association (ADA) (30, 31) were generally met throughout the training period. As shown in Figure 3C, only 2G exceeded the recommended range during the third week of the altitude training, while all other values remained within the suggested limits.

According to the LLSS findings, presented in Table 1, during the first two days of altitude training (D1 and D2), the athlete and guides reported symptoms such as headache, fatigue, and weakness, except for 3G on D2, who did not report any headaches. On D1, 1A and 2G reached the threshold for mild AMS (3–5 points), whereas 3G did not. Overall, 1A appeared more susceptible to developing AMS symptoms, while 3G showed less susceptibility. No symptoms were reported on D3 and D4, nor on the single assessment days in the third and fourth weeks of altitude training (W3 and W4), which may indicate effective acclimatization to altitude and good tolerance of the physical training protocol.

**Table 1:** The Lake Louise Symptom Score (LSS) was assessed at eight designated time points: D1, D2, D3, and D4 (first four days at altitude), W3 and W4 (third and fourth weeks of altitude training), and TZ1 and TZ3 (training zones 1 and 3).

Symptoms	1A	2G	3G	Symptoms	1A	2G	3G
**D1**				**D2**			
Headache	2	2	1	Headache	1	1	0
Gastrointestinal symptoms	0	0	0	Gastrointestinal symptoms	0	0	0
Fatigue and/or weakness	2	1	1	Fatigue and/or weakness	1	1	1
Dizziness/lightheadedness	0	0	0	Dizziness/lightheadedness	0	0	0
Score	4	3	2	Score	2	2	1
**D3**				**D4**			
Headache	0	0	0	Headache	0	0	0
Gastrointestinal symptoms	0	0	0	Gastrointestinal symptoms	0	0	0
Fatigue and/or weakness	0	0	0	Fatigue and/or weakness	0	0	0
Dizziness/lightheadedness	0	0	0	Dizziness/lightheadedness	0	0	0
Score	0	0	0	Score	0	0	0
**W3**				**W4**			
Headache	0	0	0	Headache	0	0	0
Gastrointestinal symptoms	0	0	0	Gastrointestinal symptoms	0	0	0
Fatigue and/or weakness	0	0	0	Fatigue and/or weakness	0	0	0
Dizziness/lightheadedness	0	0	0	Dizziness/lightheadedness	0	0	0
Score	0	0	0	Score	0	0	0
**TZ1**				**TZ3**			
Headache	0	0	0	Headache	0	0	0
Gastrointestinal symptoms	0	0	0	Gastrointestinal symptoms	0	0	0
Fatigue and/or weakness	0	0	0	Fatigue and/or weakness	2	2	1
Dizziness/lightheadedness	0	0	0	Dizziness/lightheadedness	0	0	0
Score	0	0	0	Score	2	2	1

Scores represent the sum of reported symptoms (headache, gastrointestinal symptoms, fatigue/weakness, dizziness/lightheadedness) for each training participant (1A, 2G, 3G). Each symptom was rated from 0 (absent) to 3 (severe). Total scores indicate AMS severity: 3 to 5 mild, 6 to 9 moderate, 10 to 12 severe, with headache required for diagnosis.

As illustrated in Table 2 (ESS), 2G exhibited a total score below the cut-off point for excessive daytime sleepiness, while 1A and 3G demonstrated values above the cut-off point. However, none of the athletes and guides obtained a score above 16, which is indicative of severe sleepiness. According to the findings presented in Table 2 (PSQI), Subjects 1A and 2G exhibited optimal sleep quality, whereas subject 3G demonstrated suboptimal sleep quality. However, none of these subjects demonstrated results that exceeded the established cut-off point for sleep disorders, and the scale score remained constant at both assessment time points.

**Table 2:** Results of the Epworth Sleepiness Scale (ESS) and Pittsburgh Sleep Quality Index (PSQI) for participants 1A, 2G, and 3G during the pre-altitude period (pre) and post-altitude (post).

ESS: how likely are you to dose off or fall asleep during the following situations?	1A		2G		3G	
	Pre	Post	Pre	Post	Pre	Post
Sitting and reading	2	2	1	1	0	0
Watching TV	3	3	1	1	2	2
Sitting, inactive in a public place	0	0	1	1	0	0
As a passenger in a car for an hour without a break	2	2	3	3	2	2
Lying down to rest in the afternoon when circumstances permit	3	3	3	3	3	3
Sitting and talking to someone	0	0	0	0	0	0
Sitting quietly after a lunch without alcohol	3	3	0	0	3	3
In a car, while stopped for a few minutes in the traffic	0	0	0	0	0	0
Total	13	13	9	9	10	10

PSQI: Components	1A		2G		3G	
	Pre	Post	Pre	Post	Pre	Post
Subjective sleep quality	0	0	0	0	1	1
Sleep latency	0	0	0	0	0	0
Sleep duration	1	1	1	1	1	1
Sleep efficiency	0	0	0	0	1	1
Sleep disturbance	1	1	1	1	1	1
Use of sleep medication	0	0	0	0	0	0
Daytime dysfunction	1	1	1	1	1	1
Global PSQI Score:	3	3	1	1	5	5

Global PSQI score: 0 to 4 good sleep quality, 5 to 10 poor sleep quality, >10 disordered sleep quality. ESS: >10 excessive daytime sleepiness, >16 severe daytime sleepiness.

During the four-week altitude training period, the athlete and his guides reported a tendency to retire for the evening 30 to 60 min earlier and to rise approximately 30 min earlier, a pattern not observed prior to training. In addition, they established a daily routine that included at least 1 h of afternoon sleep following lunch. This adjustment can be attributed to the physical training in altitude regimen, which required a considerable level of commitment. They aimed to ensure sufficient time for both proper nutrition and rest to optimize the benefits of the training period. Importantly, no athlete reported excessive sleepiness, and all indicated that their sleep duration was adequate.

## 4 Discussion

The current case report described a record-breaking Paralympic athlete and his two guides during 4 weeks of training program at moderate altitude with respect to their nutritional profile, body mass, sleep quality, and symptoms of AMS. To the best of our knowledge, this is the first case report to describe these parameters in Paralympic athletes exposed to 30 days of moderate altitude training on the LHTH regime. According to the literature, altitude training protocols are effective ways to improve the performance of athletes at sea level (4, 8). However, there are risks associated with their application, so it is necessary to monitor aspects that could potentially affect the health and performance of these individuals to ensure that the benefits of training outweigh the risks (9). Therefore, this case study can help bridge the gap between science and practice.

### 4.1 Body mass and energy balance

Exposure to altitude can increase basal metabolic rate by 400 to 600 kcal/day, reduce appetite (32), decrease caloric intake, and increase fluid loss, all of which may contribute to weight loss (33). A review of the existing literature indicates that most studies attribute this to an inadequate energy balance (34). Therefore, increasing food intake in the early days of exposure may help prevent weight loss (35). In the present case report, athletes’ body mass remained stable, with intra-individual variation ranging from −2.74 to +1.50%. These findings are consistent with those reported in elite cyclists training for 21 days at moderate altitude, who showed changes between −1.34 and +0.99% in body mass (36). In the present study, meals were prepared daily by a professional chef under the supervision of a sports nutritionist to replicate the athletes’ cultural diets. This approach helped address common nutritional challenges faced when traveling with athletes, such as cultural and religious considerations, social preferences, special dietary needs, and food intolerances and/or allergies (37).

### 4.2 Macronutrient intake

In the context of altitude expeditions, nutritional recommendations emphasize prioritizing carbohydrates as the main energy source, maintaining protein balance, and ensuring adequate hydration (34). Acute hypoxic exposure during steady-state aerobic exercise suppresses exogenous carbohydrate oxidation, suggesting that traditional guidelines may not fully meet early metabolic demands. However, this effect seems to normalize after acclimatization (38). Consuming carbohydrates 30 min before hypoxia appears beneficial, likely due to enhanced oxygen delivery, supported by increases in heart rate, ventilation, and oxygen saturation (39). Carbohydrate supplementation or increased intake of this macronutrient appears to be beneficial for maintaining or restoring glycogen stores and improving exercise tolerance (40), which can enhance performance in medium and long duration sports modalities or when athletes perform more than one training session per day. Moreover, adequate carbohydrate intake may help reduce peripheral fatigue, particularly during high intensity, short duration efforts such as the 100 m sprint in athletics. Therefore, meeting the athlete’s nutritional requirements, especially under hypoxic conditions, is crucial and seems to be associated with improved exercise tolerance, positively influencing physiological adaptations and overall performance (40).

Considering the ACSM recommendations for protein intake in athletes (1.2–2.2 g·kg^−1^·day^−1^) (29), it can be stated that, across all analyzed weeks, the athlete and his guides reported values within or above the recommended range. Evidence suggests that an intake of approximately 1. g·kg^−1^·day^−1^, may be beneficial for maintaining fat-free mass, particularly in situations of negative energy balance, as may occur under altitude conditions (41). In this context, although body composition was not assessed before and after altitude exposure, preventing confirmation of potential changes in lean mass, no major variations in body weight were observed. Furthermore, a high protein intake may increase satiety, potentially hindering the achievement of total energy requirements and leading to a reduction in carbohydrate intake, which could negatively affect glycogen levels (41). This hypothesis may help explain the findings observed in the present case study. Furthermore, athletes may follow restrictive diets, requiring attention to minimal lipid intake, which can negatively affect hormonal balance, absorption of fat-soluble vitamins, neural health, and muscle energy needs (31).

### 4.3 AMS symptoms and acclimatization

The present case study, applying the 2018 Lake Louise Acute Mountain Sickness Score (12), found symptoms such as headaches and fatigue during the first two days after arriving at moderate altitude. This finding emphasizes the need to reduce training intensity in the first days of exposure (8). A study of 38 individuals, 19 of whom were endurance-trained and 19 untrained, found symptoms of AMS in the first few days of exposure. The hypothesis proposed to explain the manifestation of symptoms during this period suggests that it may be attributable to an increase in resting metabolic rate and enhanced parasympathetic activity (42). A substantial degree of individual variability has been observed in the effects of exposure to altitude, and individuals may exhibit varying degrees of susceptibility to developing acute mountain sickness (AMS). In view of this, it has been posited that individuals exhibiting a diminished ventilatory response to hypoxia may be more susceptible to contracting this illness (43). For athletes exposed to altitude, monitoring for AMS should begin within 24 to 48 h after arrival, and interventions should be implemented according to the severity of the condition. In cases of mild AMS, as observed on D1 in the present study, training intensity should be reduced and recovery periods extended, as was applied in our protocol (44).

### 4.4 Sleep quality and cognitive aspects

The decrease in oxygen partial pressure, combined with partial or total sleep deprivation and biological adjustments induced by altitude exposure, can negatively affect cognitive performance by impairing attention, decision-making, and mood, as well as increasing fatigue and reducing neuropsychological function (45, 46). A study conducted with 40 volunteers reported that 13 h of hypoxia reduced sleep efficiency and consequently impaired mood and reaction time after 28 h. Interestingly, these effects were reversed in participants who performed acute exercise at 50% of VO₂peak under hypoxia (46), with similar findings observed for moderate-intensity exercise in both normoxia and hypoxia (47). Pirlot et al. (48) also found that altitude training improved psychophysiological responses during early acclimatization at 2,800 meters. Considering that the present study followed a progressive acclimatization protocol, gradually increasing training intensity and volume while maintaining low-to-moderate intensities during the first week at altitude, this may explain the absence of significant changes in cognitive or sleep-related questionnaires between pre- and post-altitude assessments.

Research conducted with visually impaired individuals has demonstrated a higher prevalence of sleep disorders in this demographic compared to the general population (49, 50), indicating a heightened necessity for sleep monitoring in this population. In the present case report, only one of the guides exhibited poor sleep quality according to the Pittsburgh questionnaire. However, an investigation in Paralympic athletes in normoxia observed that excessive daytime sleepiness affected more than half of the sample (51). Pedlar et al. (52) propose that the acclimatization to hypoxia through the utilization of a normobaric hypoxia tent, in conjunction with an individualized assessment, could serve as a method for predicting the impact of these conditions on sleep quality and for identifying athletes who may be susceptible to poor sleep quality.

Furthermore, according to the literature, 50% of totally blind individuals without light perception present with non-24-h sleep–wake disorder, a condition uncommon in sighted individuals, characterized by alternating episodes of insomnia and daytime sleepiness interspersed with asymptomatic periods (53). This disorder leads to circadian misalignment, resulting in hormonal dysregulation and potentially causing mood disturbances, loss of appetite, and gastrointestinal issues. Therefore, investigating sleep disorders in blind individuals becomes essential (54), as exemplified by the athlete in this case study.

In a study of 479 athletes, 52% scored ≥5 on the PSQI, raising questions about the suitability of this instrument for assessing sleep quality in athletic populations, as it may overestimate sleep problems (55). In the present case study, only one guide scored 5, which may reflect factors suggested in previous research, including caffeine intake (56), training schedules, sport-related stress and anxiety, social media use, and other lifestyle or environmental influences (55). Furthermore, 28% of athletes in a sample of 175 exhibited ESS scores ≥10, indicating substantial daytime sleepiness (57), consistent with the athlete (ESS = 13) and one guide (ESS = 10) in this study. Considering this, it may be valuable to provide athletes with guidance aimed at optimizing total sleep duration, sleep timing, and sleep quality. Strategies could include incorporating naps and extended nocturnal sleep, practicing sleep hygiene, engaging in mindfulness, and limiting the use of electronic devices before bedtime, all of which have been suggested to enhance both physical and cognitive performance (58).

### 4.5 Strengths and limitations

Although the case study has advanced knowledge in research on elite athletes, including a Paralympic, by integrating different approaches to investigate the physical training at altitude, some limitations can be considered.

First, it is important to acknowledge that the equations used to estimate basal metabolic rate and total energy expenditure provide only approximate values, which may fluctuate according to variations in training intensity and duration over time. The influence of these factors under hypoxic conditions remains uncertain, and no laboratory assessments or additional objective measurements beyond body mass were conducted. Future investigations should address this limitation by employing indirect calorimetry or other objective techniques. Another limitation concerns the potential underreporting associated with self-reported questionnaires, which rely on the honesty and memory of participants and can be influenced by contextual and social factors. Understanding individual characteristics is essential to ensure data reliability (59). Nevertheless, it is worth noting that questionnaires can be easily implemented by coaches and serve as valuable complementary tools for monitoring variables that may affect training outcomes. Furthermore, ventilatory and hematological parameters were not evaluated, which could have provided deeper insight into individual physiological responses to altitude exposure. Future studies should incorporate these assessments to better elucidate interindividual variability and adaptation mechanisms associating with nutritional and sleep quality.

Finally, it is crucial to emphasize the need for additional research to develop evidence-based nutritional and sleep recommendations for training in hypoxic environments, including in Paralympic context. Expanding the sample size would enhance data reproducibility and generalizability, enabling robust statistical analyses. However, this case study centers on a Paralympic world record holder and his guide, whose exceptional achievements offer unique insights into elite athletic performance. These findings also highlight critical knowledge gaps that must be addressed to ensure the effective and safe implementation of altitude training protocols.

## 5 Conclusion

This case study suggests that exposure to moderate-altitude training at 2,150 m, under the LHTH regimen, appeared to influence the food intake of elite athletes, even without changes in appetite. However, the program did not appear to affect sleep quality or body mass. Symptoms of acute mountain sickness were observed only in the first day at altitude, corroborating the importance of monitoring training intensity and recovery during the initial days of the LHTH regimen. Our findings also suggested that carbohydrate intake was insufficient relative to the demands of 30 days of physical training at moderate altitude. Overall, this case report reinforces the need to monitor nutritional intake, body mass, sleep quality, and subjective fatigue throughout altitude training.

In summary, these findings underscore the importance of systematically monitoring nutrition, body mass, sleep quality, and AMS symptoms during altitude training, providing practical guidance for coaches and supporting optimal preparation of elite athletes.

## Data Availability

The raw data supporting the conclusions of this article will be made available by the authors, without undue reservation.
